# Treatment of Apathy in Parkinson’s Disease and Implications for Underlying Pathophysiology

**DOI:** 10.3390/jcm13082216

**Published:** 2024-04-11

**Authors:** Senan Maher, Eoghan Donlon, Gerard Mullane, Richard Walsh, Tim Lynch, Conor Fearon

**Affiliations:** 1 Dublin Neurological Institute, Mater Misericordiae Hospital, D07 W7XF Dublin, Ireland; 2 Academic Unit of Neurology, School of Medicine, Trinity College Dublin, D02 R590 Dublin, Ireland; 3 School of Medicine, University College Dublin, D04 V1W8 Dublin, Ireland

**Keywords:** apathy, Parkinsons, Parkinson’s disease

## Abstract

Apathy is a prevalent and highly debilitating non-motor symptom of Parkinson’s disease (PD) that is often overlooked in clinical practice due to its subtle nature. This review aims to provide a comprehensive overview of the current evidence for the treatment of apathy in PD, highlighting recent advancements and emerging therapeutic avenues. In this review, we analyse a diverse array of treatment strategies for apathy in PD, including pharmacological interventions, non-pharmacological approaches, and emerging neuromodulation techniques. We evaluate the efficacy, safety, and limitations of established pharmacotherapies, such as dopaminergic agents, antidepressants, and cognitive enhancers. Additionally, we examine the promising role of non-pharmacological interventions, encompassing psychotherapies and behavioural interventions, in ameliorating apathetic symptoms. Furthermore, this review explores the effects of neuromodulation techniques on apathy, including the modulation of apathy via deep brain stimulation and emerging data on the potential influence of transcranial magnetic stimulation (TMS) on apathy in PD. Ultimately, a deeper understanding of effective treatment strategies for apathy has the potential to significantly improve the quality of life and overall well-being of individuals living with PD.

## 1. Introduction

Apathy is a syndrome consisting of diminished initiative, interest, and emotional responsiveness that is exhibited by approximately 40% of patients with Parkinson’s disease (PD) [1,2,3]. It is diagnosed when observable features such as withdrawal from daily activities and decreased spontaneous emotion emerge in the absence of worsening motor symptoms, psychiatric illness, or physical disability that would otherwise explain them [4]. Parkinsons disease is a progressive neurodegenerative disorder caused by cell loss in the substantia nigra, which supplies dopaminergic innervation to the basal ganglia. In Parkinson’s disease, apathy is associated with decreased quality of life and increased caregiver burden, making it an important therapeutic target [5,6].

The neurobiology of apathy has not been fully elucidated, but it is believed to be caused by dysfunction in brain networks related to motivated behaviour. Current models of motivated behaviour suggest the existence of both a value system and a motor system, which are under the influence of mesolimbic projections [7]. The value system, which comprises the ventromedial prefrontal cortex (vmPFC), ventral striatum, and rostral caudate, determines the cost/benefit of ongoing and potential actions. This information is then conveyed via a mediator system to a motor system, which carries out actions determined to be beneficial. This component includes the posterior mid-cingulate cortex, dorsal striatum, and supplementary motor area. The mediator system linking the two likely consists of areas of the ventral striatum and anterior cingulate, areas that are strongly influenced by dopaminergic projections from the midbrain. The observable clinical syndrome of apathy can be associated with the destruction of any of these areas, though the pattern of neuronal loss varies with specific diseases [8]. Within the context of PD specifically, many of these structures become hypofunctional or atrophied in the context of mesocorticolimbic denervation [9].

As a psychological construct, apathy has three sub-domains: emotional, cognitive, and behavioural [10]. Levy and Dubois proposed that apathy subdomains map onto observable apathy subsyndromes and that these subsyndromes have specific anatomical correlates within the network of motivated behaviour [11]. Emotional-affective apathy is characterised by emotional indifference in the face of pleasurable events and stimuli. Patients affected by this subtype of apathy will appear emotionally blunted in the absence of other depressive symptoms such as sadness, guilt, or feelings of worthlessness. This subtype is linked anatomically to the hypofunction of the orbito-medial Prefrontal Cortex (omPFC). Auto-activation deficit is a tendency to remain in a state of inertia without external stimulation. Affected patients need prompting to attend to activities including basic self-care or beloved hobbies but will report experiencing enjoyment once they are engaged. This subsyndrome arises in the context of damage to the medial PFC, basal ganglia, or globus pallidus. Finally, cognitive apathy is characterized by an inability to plan complex actions. Patients with this subtype of apathy will exhibit comorbid executive dysfunction in neuropsychological tests. It is usually related to lesions of the dorsolateral PFC. Furthermore, executive dysfunction is associated with apathy but not depression, allowing for further differentiation [12].

In a comprehensive review, Pagonabarraga et al. provided robust support for Levy and Dubois’ subtypes within the context of PD. Additionally, they proposed that account for apathy subtypes may help guide the choice of treatment. Cognitive apathy may be best treated with cholinesterase inhibitors. Patients with a predominantly auto-activation type of deficit may attain the greatest benefit from dopamine agonists. Emotional-affective apathy ought to be targeted with dopamine agonists, methylphenidate, or serotonergic agents [9]. Some studies have investigated which apathy sub-domains are most prevalent in PD. Current evidence suggests that emotional-affective apathy is less prevalent in PD than auto-activation deficit and cognitive apathy [11,13,14]. The evidence linking apathy subtypes to specific structures and therapies continues to accumulate rapidly. However, in current practice, apathy is still treated as a unidimensional construct. Treatment based on apathy subtypes may become established in routine practice in the future [15].

Regardless of the complexities of the underlying pathophysiology, at a clinical level, apathy is a highly debilitating and prevalent symptom in PD [6]. It can emerge at all disease stages, often exists without comorbid depression, and is associated with an earlier onset of motor symptoms and cognitive impairment [2,16]. Apathy in PD is associated with lower premorbid educational attainment, depression, cognitive impairment, a longer disease course, and lower dopamine agonist (DA) doses, among other things [17,18,19]. Apathy is more frequent in patients with Rapid Eye Movement Sleep Behaviour Disorder (REMSBD), and this may also constitute a significant risk factor [20]. It may be that poor sleep quality is causally linked to apathy, or it may be that both apathy and REMSBD emerge in the context of serotonergic dysfunction in early PD [21,22,23]. Regardless, at the clinical level, apathy is under-recognized in neurological practice and frequently misdiagnosed, leading to frustration for patients and clinicians alike [24]. When it is detected, physicians are faced with an array of therapeutic options.

These therapies can be broadly divided into two groups: pharmacological and non-pharmacological. The most extensively researched and commonly prescribed pharmacological treatments for apathy are dopaminergic agents (e.g., rotigotine) and cholinesterase inhibitors (e.g., rivastigmine) [1,25]. However, the list of trialled therapies continues to grow, and recently published studies have examined vortioxetine [26] and the experimental compound pirepemat [27]. Non-pharmacological therapies that have shown specific benefits for apathy in PD include exercise therapy and mindfulness. Within the wider context of neurodegenerative diseases, however, numerous non-pharmacological therapies have shown promise and may have a potential role in apathy in PD [28].

In the continued absence of apathy-specific treatment guidelines, this review aims to summarise the current knowledge of available therapies for this troubling symptom while linking them to current models of the underlying pathobiology of apathy.

## 2. Pharmacological Therapy for Apathy

### 2.1. Cholinesterase Inhibitors

Rivastigmine has shown efficacy for treating PD-related apathy in addition to patient quality of life and caregiver strain in randomized controlled trials (RCTs) [29,30]. In one study, a significant difference in the Lille Apathy Rating Scale (LARS) was seen at six months following initiation of rivastigmine treatment, but this difference was not sustained at 18 months [29]. In another study on patients with Lewy Body Dementia (LBD), the authors found that rivastigmine led to a reduction in apathy when assessed as part of the Neuropsychiatric Inventory (NPI) at 20 weeks. Apathy was the symptom that showed the greatest improvement among a number of neuropsychiatric symptoms [30].

Acetylcholine is involved in the modulation of the mesolimbic projections, which are themselves key modulators of the entire system of motivated behaviour [31,32]. In other neurodegenerative disorders, cholinesterase inhibitors may improve apathy via improving cognitive function and planning, a function that is localized in part in the dorsolateral prefrontal cortex (dlPFC) [33]. Therefore, in PD patients with cognitive apathy, who have difficulty formulating a plan of action, cholinesterase inhibitors should be considered [9].

On a practical level, the use of cholinesterase inhibitors should be avoided for patients with bradycardia or recurrent syncope [34]. Cholinesterase inhibitors may also precipitate urinary retention in some patients and must therefore be prescribed with caution [35].

### 2.2. Dopamine Agonists

Several studies have examined the effect of dopamine agonists on apathy in PD [36]. In an assessment of the effect of rotigotine on Nonmotor Symptom Scale (NMSS) scores, while the overall improvement in scores was not significant at 12 weeks, the improvement in the apathy subdomain was significant. This result must be interpreted with caution as the NMSS has not yet been shown to have convergent validity with respect to other apathy measures in PD and, in fact, may not have sufficient divergent validity with respect to depression [37]. Indeed, a follow up RCT using the LARS as a primary outcome measure did not show a significant effect [38]. However, a shorter-duration study demonstrated a significant benefit associated with rotigotine when apathy was assessed as a secondary outcome using the apathy scale. Despite this mixed evidence base, dopamine agonists remain a commonly recommended therapy for apathy in PD [39,40,41].

The mechanism by which dopamine might ameliorate apathy has not been elucidated fully. The traditional view of dopamine as a “pleasurable reward chemical” has been challenged by the observation that dopamine-depleted mice appear to exhibit their usual hedonic behaviours when exposed to a pleasurable stimulus [42]. However, depletion appears to make mice less likely to expend effort to obtain high-quality rewards, even if they find such rewards enjoyable [43]. It may therefore be that the mechanism of dopamine repletion in treating apathy consists of facilitating the ability to expend effort in order to carry out pleasurable behaviours; however, this is speculative. Given the importance of phasic dopaminergic stimulation upon receiving a reward, one might expect that immediate-release formulations could have a distinct effect on apathy compared with more prolonged release formulations such as rotigotine; however, this has not been studied to date. It is not clear whether or not dopamine agonists are more efficacious than levodopa in treating apathy. Dopamine agonists certainly are more likely to induce impulse control disorder than levodopa, so it may be the case that they have a differential effect on apathy also. The difference in effect is thought to be explained by differential affinity for dopamine receptors, with dopamine agonists having greater affinity for D3 receptors than levodopa [44,45]. In a validated lesional model of PD, only D3 agonism was shown to improve the motivational deficits induced by the destruction of the substantia nigra [46]. Hence, PD patients with apathy predominantly defined by an auto-activation deficit should be preferentially trialled on dopamine agonists [16,47].

Co-prescription of dopamine agonists to patients with established PD may necessitate down-titration of their levodopa dose. Patients on dopamine agonists may experience central dopaminergic side effects such as dyskinesia, nausea, and hallucinations along with peripheral dopaminergic side effects such as hypotension and drowsiness [48].

### 2.3. SSRIs

Only one study assessed the association between Selective Serotonin Reuptake Inhibitor (SSRI) treatment and apathy scores with respect to PD patients. In total, 55 patients were randomized to receive either open-label SSRIs (paroxetine or escitalopram) or an SNRI (duloxetine) [49]. This study was not placebo-controlled, and both groups showed modest but not statistically significant reductions in their apathy scores. Trials assessing SSRIs as a treatment for apathy pertaining to other neurodegenerative diseases have mostly failed to demonstrate a significant effect [1]. However, one trial involving patients with Alzheimer’s disease did show a significant reduction in apathy when citalopram was added to memantine therapy [50]. In PD, SSRIs are certainly helpful in treating depression, which is often a comorbidity of (but distinct from) apathy [51]. Given the symptom overlap between apathy and depression, apathy scores may appear to improve somewhat if measured with a scale with low divergent validity with respect to depression [52].

SSRIs may be best targeted towards those with emotional-affective apathy that has been mapped to the mesocorticolimbic circuits comprising “the reward pathway” [16]. Serotonin uptake reduction along this pathway has been shown to be proportional to the degree of apathy (as measured using the LARS), suggesting that therapies that increase serotonin may play a role.

SSRIs are generally well tolerated by patients. Side effects include QTc prolongation, sexual dysfunction, and hyponatremia. However, particular caution must be taken when prescribing SSRIs to a patient with PD as they may also have been prescribed a MAO-B inhibitor such as selegiline as a co-prescription, potentially precipitating serotonin syndrome [53].

### 2.4. Memantine

One trial assessed the effect of memantine on apathy in PD as measured using the NPI [54]. The trial did not demonstrate a significant effect compared with a placebo, although a significant placebo effect was noted. Individual studies on Alzheimer’s disease have demonstrated positive results with respect to apathy; however, a meta-analysis of available data concluded that there was no effect [55].

The mechanisms by which memantine might ease apathy are currently unknown. Memantine is an N-methyl-D-aspartate (NMDA) channel antagonist that can improve cognitive function in cases of dementia [56]. Memantine may exert its effects on apathy indirectly via improvements in cognitive function; however, this is speculative. Memantine is generally well tolerated by people with PD and continues to be used to target cognitive impairment, although high-quality evidence for its efficacy in this domain is also lacking [57]. The dose must be adjusted in the case of renal impairment. Commonly reported side effects include dizziness and headache [58].

### 2.5. Methylphenidate

Methylphenidate leads to increases in motivated action even in non-clinical populations and is considered promising for the treatment of apathy in disorders such as PD and Alzheimer’s disease [59]. In one study, apathy was assessed as a secondary outcome in a trial of methylphenidate for treating gait freezing and instability in PD [60]. It showed a non-significant change in LARS apathy scores at 3 months; however, the researchers noted that seven of the patients assigned to methylphenidate showed a significant improvement. Further case-report data have shown a significant improvement in apathy for patients with PD [61]. Methylphenidate is a dopamine and catecholamine reuptake inhibitor that increases concentrations of these neurotransmitters in both the striatum and medial PFC, key areas in the network of motivated behaviour [62]. However, there appears to be a U-shaped dose–response relationship, with both very low concentrations of catecholamines and very high concentrations being associated with decreased prefrontal cortical connectivity [63]. Additionally, methylphenidate may improve neuronal integrity. In one case, such an improvement in neuronal integrity in the frontal lobes was demonstrated to be accompanied by clinical improvement in apathetic symptoms [64]. Further trials on the use of methylphenidate for treating apathy in PD are planned [65]. On a practical level, clinicians must exercise caution when prescribing methylphenidate to patients with PD. It may not be prescribed concurrently with monoamine oxidase inhibitors (MAOis), and it must also be avoided in cases of severe hypertension, which may apply for PD patients with autonomic dysfunction [65]. Methylphenidate is also known to infrequently precipitate psychosis in adolescent populations, and clinicians must therefore be cautious when prescribing it to PD patients with a history of psychotic symptoms.

### 2.6. Vortioxetine

A recent single-arm open-label study, the VOPARK study, evaluated vortioxetine as a therapy for depressive symptoms suffered by PD patients who concurrently met diagnostic criteria for major depressive disorder. The investigators found that as well as improving mood, there was a significant effect on apathy as measured using the apathy scale [26]. While we could not find any further studies that evaluate the effect of vortioxetine on apathy, one trial found a significant short-term effect on anhedonia. There is significant overlap between apathy and depression; in particular, it would appear that apathy and the depressive subdomain of “anhedonia” may not be entirely distinct and could share common neural mechanisms [66]. Further studies have demonstrated that vortioxetine may have greater efficacy than other SSRIs and SNRIs with respect to the apathy-related construct of “emotional blunting” [67].

Vortioxetine has a unique pharmacological profile. In addition to its activity as a serotonergic agent, it is also known to be active in dopaminergic and catecholaminergic neurotransmission [68]. While it is not known how vortioxetine may exert its effect within the network of motivated behaviour, it may be active at multiple sites given its diverse psychopharmacological profile. Additionally, we may speculate that the observed effects on the related constructs of anhedonia and emotional blunting imply that it would be prudent to prioritize vortioxetine for use in patients with emotional-affective apathy in future studies.

Vortioxetine is generally well tolerated and appears to have fewer sexual side effects than other antidepressants [69]. As with SSRIs, vortioxetine may not be prescribed with MAOIs due to the risk of serotonin syndrome.

### 2.7. Pirepemat (IRL-752)

Pirepemat is a novel small-molecule compound classified as a cortical enhancer, demonstrating regioselective potentiation of cortical norepinephrine, dopamine, and acetylcholine [70]. A phase 2b RCT demonstrated the safety and tolerability of pirepemat for treating PD, with a significant reduction in apathy severity scores as measured using the NPI at 4 weeks [27]. The authors speculate that this therapeutic effect may be mediated via effects on cortical norepinephrine. The simultaneous improvement in cognitive function might suggest that pirepemat plays a role in cognitive apathy. Its role in other apathy subtypes is yet to be clarified. Pirepemat was generally well tolerated, although transient elevations of liver enzyme levels were seen in some patients. Further trials of pirepemat are planned [71].

### 2.8. Other Pharmacological Therapies Trialled for PD

MAOIs have so far failed to demonstrate a significant effect on apathy in PD, although a number of studies showed a trend towards improvement, which may warrant further exploration [72,73]. Atomoxetine therapy for treating PD showed a trend towards improved cognitive function but did not result in a significant improvement in neuropsychiatric symptoms, including apathy [74].

### 2.9. Conclusions

There are numerous available pharmacological options for the treatment of apathy in PD; however, none have shown a clear advantage over the rest. It may be that each treatment is best targeted towards a specific apathy subtype. Future trials that select patients based upon the pathophysiological mechanism of apathy may demonstrate more success.

## 3. Non-Pharmacological Therapy for Apathy

A broad range of nonpharmacological therapies have been trialled to treat apathy across the spectrum of neurodegenerative disorders [28]. In PD specifically, both mindfulness interventions and a variety of exercise programmes may have benefits [75]. However, s patient’s ability to engage in exercise interventions is dependent upon their mobility, while impaired cognitive ability may limit meaningful involvement in mindfulness programmes. Recent research has focused on the effect of music therapy and transcranial magnetic stimulation, which hypothetically could be used to treat patients with more advanced disease.

### 3.1. Mindfulness Interventions

Mindfulness has been defined as “paying attention in a particular way, on purpose, in the present moment, and nonjudgmentally” [76]. Mindfulness-based interventions seek to cultivate this skill among patients and integrate it into their daily lives. In practice, these interventions often take the form of either Mindfulness-Based Stress Reduction (MBSR) or Mindfulness-Based Cognitive Therapy (MBCT) [77].

One trial recruited 34 PD patients and several of their carers to partake in an eight-week pilot study in which they delivered a mindfulness programme modelled on MBSR [78]. The intervention consisted of once-weekly group sessions of mindfulness education and practice followed by a half-day mindfulness retreat. This pilot study did not demonstrate a significant improvement in apathy as measured using the apathy scale and, furthermore, suffered from a high attrition rate (25%). Another pilot study examined a group-based mindfulness training intervention involving 14 PD patients, but no significant difference in apathy levels was demonstrated in their post-intervention assessment [79].

From a neurobiological perspective, mindfulness meditation is most commonly linked with changes in structure and activity in the anterior cingulate cortex [80]. This area is one of the primary connecting pathways between subcortical structures and wider cortical areas in the network of motivated behaviour; additionally, hypofunction here is associated with all major apathy subsyndromes [7,9], Mindfulness interventions may not be suitable for PD patients with cognitive impairment.

### 3.2. Exercise Interventions

Increased exercise levels among PD patients are associated with a decreased rate of decline in a number of important domains, including postural stability and mental processing speed [81]. Therapeutic exercise interventions are known to promote neuroplasticity and neuronal repair in PD patients [82]. One study demonstrated that an exercise intervention improved dopaminergic function in nigrostriatal and mesolimbic pathways when assessed using functional neuroimaging [83]. Several studies have examined the effects of a variety of exercise-based interventions on clinical apathy scores. Some interventions, including dance interventions [84,85] and Nordic walking [86], were associated with improvements in apathy scores. However, other forms of dance [87], agility training [88], circuit training [89], boxing [90], and table tennis [91] interventions showed no differences. Technologically enhanced exercise interventions have also been trialled for use in PD and may hold promise in the treatment of apathy [92]. It is unclear why some exercise interventions appear to be effective while others do not. All the studied interventions were complex, and it can be difficult to isolate the effects of exercise itself. Many exercise interventions contain social elements and elements of behavioural activation, among others, which may have an indirect impact on apathy. On a practical level, exercise interventions can be difficult to implement in a healthcare system due to resource and staffing constraints, among others [93]. Apathy itself is also a significant barrier to participation in exercise programmes in the context of PD [94]. It may therefore be that the patients in greatest need of this therapy are the least likely to be able to engage with it.

### 3.3. Behavioural Activation

Behavioural activation therapy encourages patients to analyse their own behaviours and the environments in which they arise. Therapists help to create activity plans and schedules that will lead to encounters with positively reinforcing environments. These, in turn, will lead to more positive behaviours [95]. Unlike Cognitive Behavioural Therapy (CBT), the main focus is upon observable behaviour rather than thoughts and feelings, which are not accessible to an outside observer. One group adapted a brief behavioural activation programme that had been designed for use on depressed patients and delivered it to 27 patients with PD [96]. They found a significant effect on apathy scores as measured using the LARS, and it was sustained at 1 month post-intervention. Functional neuroimaging studies have shown increased activation of the medial prefrontal cortex in response to behavioural activation therapy [97]. This area is implicated in both auto-activation deficits and emotional-affective apathy. However, given that behavioural activation therapy encourages the creation of environmental prompts and positively reinforcing external environments, it may be that patients with auto-activation deficits should be preferentially targeted with this therapy. Psychotherapists’ widespread use of this therapeutic approach for treating depression may suggest utility for emotional-affective apathy as well.

### 3.4. Music Therapy

A pilot study examined virtual music therapy as a treatment for apathy for patients with PD [98]. The patients participated in an online music class weekly for 12 weeks and took part in vocal exercises, rhythmic drumming, and group singing. The 16 participants presented a significant reduction in apathy scores post-intervention. This study was limited by its lack of a control group, but controlled studies on other neurodegenerative disorders have also demonstrated positive results [99]. Furthermore, music therapy may deliver benefits beyond improvement in apathy including effects on depressive and behavioural symptoms of dementia [100]. It is not yet clear how music therapy might fit into the network of motivated behaviour or if it may be targeted towards specific apathy subtypes in the future. Further controlled studies in this area are warranted.

### 3.5. Transcranial Magnetic Stimulation

High-frequency Transcranial Magnetic Stimulation (TMS) has been shown to effectively modulate excitatory cortical neuronal activity, although the neuropsychological effects of TMS remain somewhat controversial. One study investigating TMS for apathy demonstrated that TMS applied over the right dorsolateral prefrontal cortex (DLPFC) is beneficial for emotional processing in both apathetic and non-apathetic PD patients. Apathy was measured using specific event-related potentials reflecting emotional response to visual stimuli and facial expressions [101]. Dopaminergic denervation in the dorsolateral prefrontal cortex has been identified in other studies as a neural correlate for apathy in PD [102]. A recent meta-analysis of 12 randomised control trials of repetitive TMS for treating both motor and non-motor symptoms in PD demonstrated high-frequency rTMS applied to the dlPFC improved depressive symptoms compared to a placebo, but it did not specifically look at apathy as an outcome [103]. Overall, there is a growing body of literature supporting the use of TMS as an adjunct therapy for apathy for patients with neurodegenerative conditions, but the evidence regarding PD-related apathy is not as strong as it is for other conditions like Alzheimer’s disease, and further large trials are needed [104].

### 3.6. Deep Brain Stimulation

Further insights into the pathobiology of apathy may be gained from studies on PD patients undergoing deep brain stimulation (DBS), although the effect is variable. Apathy is a relatively frequent observation following deep brain stimulation of the subthalamic nucleus(STN-DBS) for PD [9]. Neurostimulation of the subthalamic nucleus (STN) mimics the best “ON” motor effects of dopaminergic therapy, and therefore dopaminergic medications are usually reduced in the early postoperative period. This complicates attempted mechanistic explanations of the observed apathy. However, a meta-analysis of apathy post-STN-DBS demonstrated a significant increase in apathy scores, and this effect was independent of tapering dopaminergic medications [105]. Anatomically, the STN has motor, associative, and limbic subdivisions, with dysfunction in associative and limbic networks thought to underlie DBS-related apathy. In one study, investigators utilised a 7T diffusion-weighted MRI to identify these STN subdivisions and their respective cortical projections. They found that in patients with postoperative apathy, active leads were more often placed in regions with a higher density of projections to associative cortical regions and lower the density of projections for motor regions [106]. An increase in apathy scores also correlates with greater dorsolateral lead placement [107]. Despite these anatomical correlations, some studies have shown no difference between apathy scores among PD patients who undergo DBS surgery and those who do not [108].

Rapid withdrawal of dopamine agonists after starting DBS can be associated with “dopamine withdrawal syndrome”. This consists of a triad of apathy, anxiety, and depression. Apathy secondary to drug withdrawal tends to occur almost immediately within days of dose reduction/drug cessation [109]. Separately, apathy may develop secondary to chronic stimulation. This often arises 4–5 months post-implantation and is thought to be due to the plastic effects of chronic stimulation [109]. The only consistently identified risk factor for postoperative apathy is the presence of preoperative non-motor fluctuations suggesting a pre-existing dysfunction of mesocortical and mesolimbic pathways. In addition to new-onset apathy, the effect of DBS on pre-existing apathy has been explored. However, the results are conflicting, and it is possible that this conflict is explained by different underlying mechanisms of the observed apathy syndrome [110].

### 3.7. Conclusions

The natural diversity of non-pharmacological therapies often makes drawing general conclusions difficult. The case for the current literature on non-pharmacological approaches for treating apathy in PD is no different. Large, well-designed studies are clearly needed, but, again, patient selection will be important to demonstrate intervention-specific effects rather than indirect effects of social engagement, which are inherent to many of these therapies. Non-pharmacological studies are limited by setup cost and can be difficult to integrate into routine practice. Further evidence of effects is needed.

## 4. Interpretation

No existing therapy has shown overwhelming evidence regarding the successful treatment of apathy in PD. The existing literature on pharmacological treatments is limited in a number of ways. Firstly, the existing scales used to measure apathy vary in their sensitivity to apathy. For example, the Unified Parkinsons Disease Rating Scale (UPDRS) was used in several studies despite its insensitivity to apathy compared with other scales [111]. Other scales, such as the apathy scale, fail to capture the multi-dimensional nature of apathy, meaning that trialled therapies cannot be evaluated with reference to the predominant subtype observed in a study [112]. Validated, multi-dimensional scales such as the Lille Apathy Rating Scale may be optimal for future studies [113]. A second limitation expressed in the existing literature is that most studies were of a short duration. Few studies assessed whether or not a therapeutic effect was sustained over a long period, and those that did reported negative results. Thirdly, many studies assessed apathy as one of numerous secondary outcomes rather than as a primary outcome. This decreases the likelihood that the observed effects reflect reality.

The literature on nonpharmacological treatments for apathy in PD is also limited. The corresponding interventions were typically complex, making it difficult to assess which elements may be helpful in treating apathy. These interventions were also often resource-intensive, meaning they may not be useful in resource-poor settings. Furthermore, many interventions required a significant amount of patient engagement, meaning they would be restricted to PD patients with relatively mild disease.

## 5. Future Directions

Several therapies, both pharmacological and nonpharmacological, have been demonstrated to be potentially suitable for apathy. Future well-designed studies may further support the integration of one or several of these therapies into routine care. The move to a more dimensional view of apathy may also be transformative. In future, therapeutic trials must be targeted towards suitable patients depending upon their apathy subtype so that appropriate medications can be trialled in groups of patients whose underlying pathophysiology should lend itself to pharmacological targeting. In this way, a move to a more personalized approach to treating apathy can be undertaken, but large studies are likely needed.

There is no evidence suggesting that DBS can be used as an effective treatment for apathy. However, emerging evidence suggests that precise targeting may improve the treatment-related apathy syndrome sometimes seen with STN DBS. Non-invasive forms of neuromodulation such as TMS show early evidence of efficacy, but larger trials are needed to verify these preliminary findings. There are a number of promising signs.

## Data Availability

No new data were created or analyzed in this study. Data sharing is not applicable to this article.
